# Efficacy and Safety of Laparoscopy for Mild and Moderate Pediatric Abdominal Trauma: A Systematic Review and Meta-Analysis

**DOI:** 10.3390/jcm11071942

**Published:** 2022-03-31

**Authors:** Yun Chul Park, Young Goun Jo, Young-Jun Ki, Wu Seong Kang, Joongsuck Kim

**Affiliations:** 1 Division of Trauma, Department of Surgery, Chonnam National University Medical School and Hospital, Chonnam National University, Gwangju 61469, Korea; kontikithor@gmail.com (Y.C.P.); thinkjo82@gmail.com (Y.G.J.); 2 Division of Acute Care Surgery, Department of Surgery, Asan Medical Center, University of Ulsan College of Medicine, Seoul 05505, Korea; kyj8302@hanmail.net; 3 Department of Trauma Surgery, Jeju Regional Trauma Center, Cheju Halla General Hospital, Jeju 63127, Korea; jsknight68@gmail.com

**Keywords:** laparoscopy, pediatric trauma, systematic review, meta-analysis

## Abstract

In this systematic review and meta-analysis, we aimed to investigate the efficacy and safety of laparoscopy for pediatric patients with abdominal trauma. Relevant articles were obtained by searching the MEDLINE PubMed, EMBASE, and Cochrane databases until 7 December 2021. Meta-analyses were performed using odds ratio (OR) for binary outcomes, standardized mean differences (SMDs) for continuous outcome measures, and overall proportion for single proportional outcomes. Nine studies examining 12,492 patients were included in our meta-analysis. Our meta-analysis showed younger age (SMD −0.47, 95% confidence interval (CI) −0.52 to −0.42), lower injury severity score (SMD −0.62, 95% CI −0.67 to −0.57), shorter hospital stay (SMD −0.55, 95% CI −0.60 to −0.50), less complications (OR 0.375, 95% CI 0.309 to 0.455), and lower mortality rate (OR 0.055, 95% CI 0.0.28 to 0.109) in the laparoscopy group compared to the laparotomy group. The majority of patients were able to avoid laparotomy (0.816, 95% CI 0.800 to 0.833). There were no missed injuries during the laparoscopic procedures in seven eligible studies. Laparoscopy for stable pediatric patients showed favorable outcomes in terms of morbidity and mortality. There were no missed injuries, and laparotomy could be avoided for the majority of patients.

## 1. Introduction

Recently, minimally invasive surgery using laparoscopy has been critical in trauma care [1]. Laparoscopy has major advantages, including minimal incisional wound resulting in less pain or less wound infection, early recovery of bowel function, and less adhesion, whereas laparotomy is the gold standard for unstable patients. Surgery aims not only to determine the injury in patients with abdominal trauma but also to treat injured abdominal organs. Therefore, the advantage of laparoscopy is emphasized in cases where non-therapeutic laparotomy can be avoided. Non-therapeutic laparotomy results in postoperative morbidities, such as unnecessary excessive incisional wounds and postoperative adhesions [2]. In a recent cohort of pediatric patients who underwent surgery, the incidence of adhesive small bowel obstruction was 12.6% even after a median follow-up of 14.7 years [3]. Therefore, minimizing adhesions via laparoscopy would be beneficial because adhesive complications can last a lifetime. Despite the advantages of laparoscopy, there have been several controversies regarding its applicability to various injured organs and its ability to detect all potential organ injuries. In general, there are two major concerns regarding pediatric patients with abdominal trauma. First, prompt management, such as an emergent laparotomy, is mandatory for patients with hemodynamic instability. Second, missed injuries, such as a bowel perforation, should be avoided. For the application of laparoscopy, we should dispel these concerns and select appropriate patients.

Recent improvements in laparoscopic skills and devices may render laparoscopy useful as a putative diagnostic or therapeutic option for patients with trauma [1]. Previous systematic reviews and meta-analyses indicated favorable results in terms of efficacy and safety for both blunt and penetrating abdominal trauma in adult patients [1,4,5,6,7]. Modern laparoscopic surgical skills have been appropriate for the operation of various organs, including the stomach, colon, and pancreas in patients with cancer, and some randomized control trials have been conducted [8,9,10]. However, previous meta-analysis regarding trauma laparoscopy reported no randomized control trials. The randomization of patients with trauma would be challenging, and the level of evidence would be inevitably low. Moreover, there have been no systematic reviews and meta-analyses regarding laparoscopy for pediatric patients. In pediatric patients, laparoscopy may be limited due to the small size of the abdominal cavity. The evidence is also limited in pediatric patients with trauma compared to adult patients likely because the incidence of pediatric trauma (<19 years old) was 16.4% among the general population, and severity was lower in pediatric than in adult patients, according to an annual report by the National Trauma Data Bank (NTDB) in the United States [11]. Nevertheless, minimally invasive surgery may be important in pediatric patients because they are considerably vulnerable to surgical insult, which is regarded as secondary damage. In the era of minimally invasive surgery, an investigation of evidence and effect size for pediatric patients is essential.

In this systematic review and meta-analysis, we aimed to investigate the efficacy and safety of laparoscopy for pediatric patients with abdominal trauma.

## 2. Materials and Methods

### 2.1. Published Study Search and Selection Criteria

This study was conducted following the Preferred Reporting Items for Systematic Reviews and Meta-Analysis [12]. The protocol of this study was registered in PROSPERO prospectively (CRD42020204044, https://www.crd.york.ac.uk/prospero, accessed on 13 August 2020). Relevant articles were obtained after searching the MEDLINE PubMed, EMBASE, and Cochrane databases until 7 December 2021. These databases were searched using the following keywords: “((trauma OR traumas OR traumatic) OR (wound OR wounds OR wounded OR injury OR injuries)) AND (((abdominal injuries) OR (abdominal injury) OR (abdomen) OR (Spleen OR splenic OR liver OR hepatic OR kidney OR renal OR diaphragm OR diaphragmatic OR pancreas OR pancreatic)) AND (laparoscopy OR laparoscopic OR (minimal invasive)) AND (pediatric OR child OR children)”. In addition, we manually searched the reference lists of relevant articles. The titles and abstracts of all the searched articles were screened for exclusion. Review articles or previous meta-analyses were also screened to identify additional eligible studies. The search results were then reviewed; the articles that investigated laparoscopy for pediatric patients with abdominal trauma were included.

Pediatric patients are defined as patients who are younger than 19 years old. The inclusion criteria for this review were as follows: (i) pediatric patients with abdominal trauma; (ii) patients who underwent laparoscopic surgery; (iii) comparison between laparoscopy and laparotomy; (iv) report of relevant outcomes, such as operative and postoperative measurements; (v) report of odds ratio (OR) or mean with standard deviation or provision of data for their calculation; (vi) single proportional data if there is no comparison between interventions. The articles that examined other diseases or adult patients, non-original research articles, or non-English-language publications were excluded.

### 2.2. Data Extraction

Data from all eligible studies were extracted by two investigators. The following data were extracted from each eligible study: name of the first author; year of publication; study location; study design; study period; number of patients analyzed; age of patients; injury severity score (ISS); name of operation; avoidance of laparotomy; missed injury; therapeutic laparoscopy; duration of hospital stay; overall complications; and mortality rate. The avoidance of laparotomy was defined as a successful laparoscopy without conversion to an open laparotomy. A laparoscopy-assisted surgery that accompanied a mini-laparotomy was regarded as a conversion. A therapeutic laparoscopy was defined as a fully laparoscopic procedure for therapeutic purposes, such as a suture or a ligation. A missed injury was defined as an occurrence of postoperative complications due to missed injury. To calculate the mean difference for meta-analysis when a median value with interquartile range was reported, normal distribution was assumed, and we calculated the mean with standard deviation [13].

### 2.3. Quality Assessment

To evaluate the risk of bias in the observational studies, we employed a tool previously used for assessing the risk of bias in non-randomized studies of interventions (ROBINS-I) [14]. All studies were independently reviewed by two investigators. Any disagreement concerning study selection and data extraction was resolved through a consensus.

### 2.4. Statistical Analysis

All statistical analyses were performed using the “meta” R package, version 4.1.1 (R foundation, Vienna, Austria). The visualizations of the risk of bias were performed using the “robvis” R package. Meta-analyses were performed using ORs for binary outcomes, standardized mean differences (SMDs) for continuous outcome measures, and an overall proportion for single proportional outcomes. To pool the proportion (complete resection, recurrence, and complications), we used logit-transformed values to avoid squeezing the variance effect [15,16]. Confidence intervals were calculated using the exact confidence limits for a binominal proportion [15]. To pool the OR for binary data and SMD, we used the inverse-variance weighing method for the meta-analysis of the outcomes. Hedges’ g was used to correct the bias of SMDs. Heterogeneity was assessed through a visual inspection of the forest plots and estimated by using I^2^ statistics and Cochran’s Q (Chi-square test) (*p* < 0.10 was considered significant). I^2^ statistics >25%, >50%, and >75% were considered to represent low, moderate, and high heterogeneity, respectively [17]. Due to the low number of eligible studies (<20), we could not assess publication bias using statistical methods (e.g., funnel plots and Egger regression test) [18].

We performed a subgroup analysis to assess heterogeneity across the studies. The effect sizes of the baseline characteristics (age and ISS), morbidity, mortality, and the quality of laparoscopic procedure were calculated according to the study-level moderator, which is the data source of this study. Institution data were defined as data from an individual institution. We generated two groups, institution data and National Trauma Database (NTDB) data, because the definition of the disease and intervention was heterogenous in each database. In addition, it is possible that some data may be duplicated because NTDB included more than 900 institutions in the United States [11]. We also conducted a sensitivity analysis by omitting each study to ensure robustness.

## 3. Results

### 3.1. Selection and Characteristics

**Figure 1:**
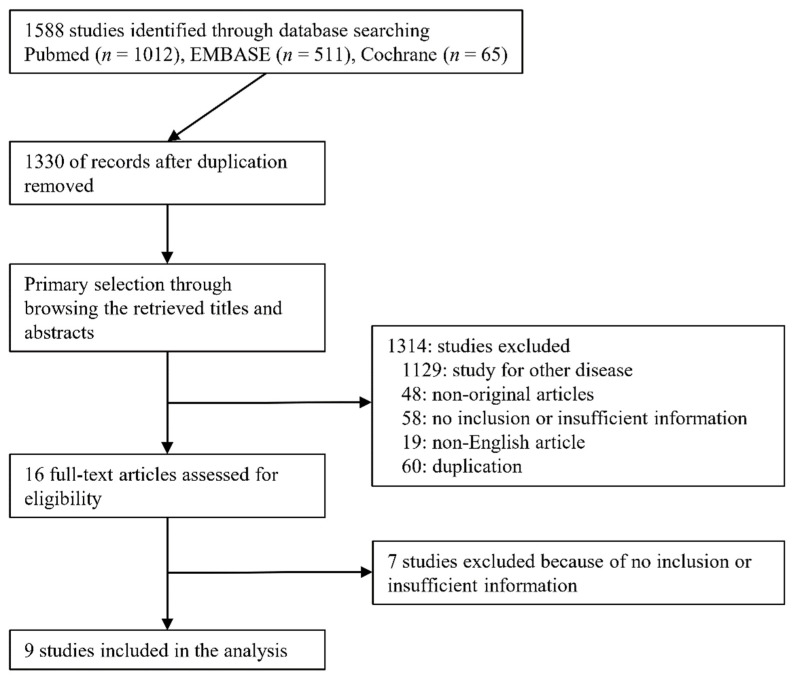
Flow diagram for the identification of eligible studies.

A total of 1588 studies were identified through the database search. Among the searched studies, 1314 were excluded because they examined other diseases (*n* = 1129), were non-original research studies (*n* = 48), did not include or had insufficient information (*n* = 58), were written in a non-English language (*n* = 19), or were duplicated (*n* = 60). Finally, nine studies [19,20,21,22,23,24,25,26,27] examining 12,492 patients were included in the present meta-analysis (Figure 1).

The detailed information of the eligible studies is summarized in Table 1. All were observational studies, and there were no randomized studies. All studies were conducted at children’s hospitals or pediatric trauma centers. Five studies were multicenter trials [20,22,23,25,27]. Evans et al. [25] reported two cohorts derived from a single institution and NTDB; hence, we enrolled both institutional data and nationwide data independently in our meta-analysis. In our search during the systematic review, we found two studies [28,29] that derived from NTDB and one study [30] that derived from the pediatric trauma quality improvement program (TQIP) database, which utilizes the infrastructure from NTDB. However, the study periods of these studies overlapped with the NTDB cohort from Evans et al., and they used relatively limited indications, which resulted in a smaller number of patients. Thus, we used only one NTDB cohort [25]. Six studies [19,20,21,22,24,25] compared laparoscopy with laparotomy, whereas three studies [23,26,27] reported only laparoscopy. One study [26] comprised only blunt trauma, and one study [27] comprised only penetrating trauma. One study [22] comprised only grade 3 pancreatic injury, and one study [26] comprised only blunt liver and spleen injury. The hemodynamic status of the laparoscopic group was stable in eight studies [19,20,21,22,24,25,26,27], whereas one study [23] did not report the hemodynamic status.

### 3.2. Comparison between Laparoscopy and Laparotomy

**Figure 2:**
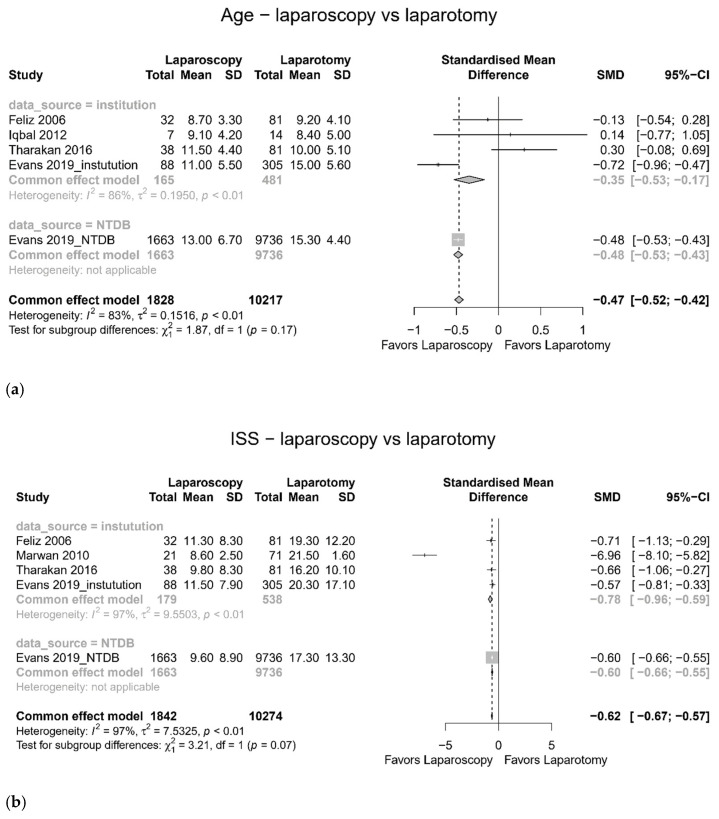

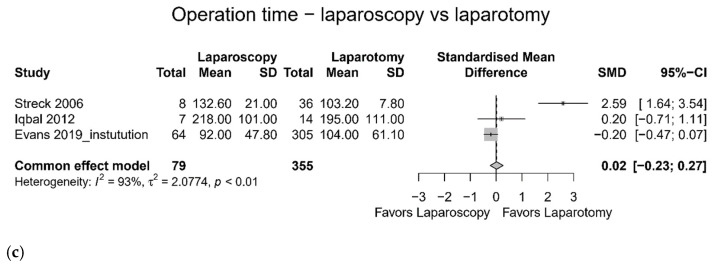
Age, injury severity score (ISS), and operation time (laparoscopy versus laparotomy). (**a**) age, (**b**) ISS, and (**c**) operation time.

**Figure 3:**
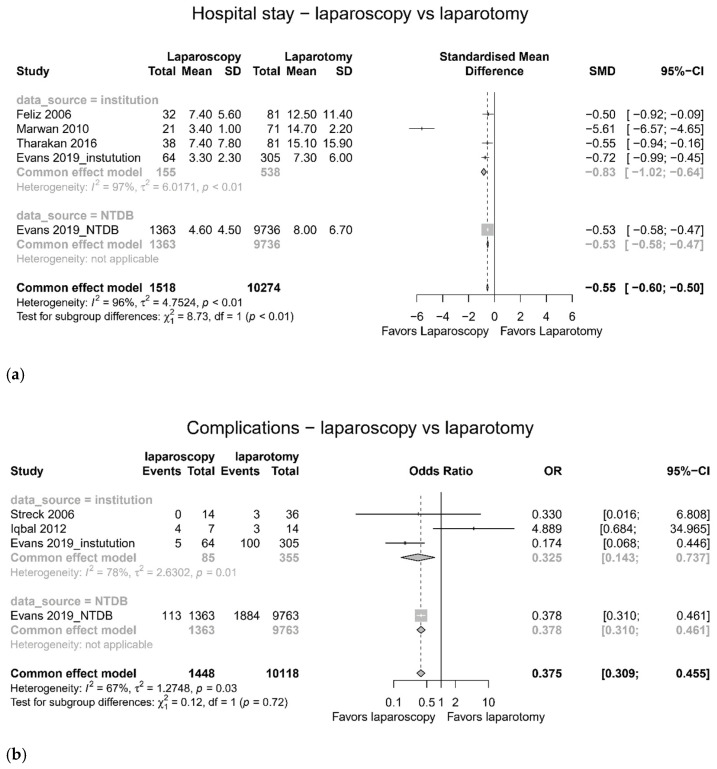

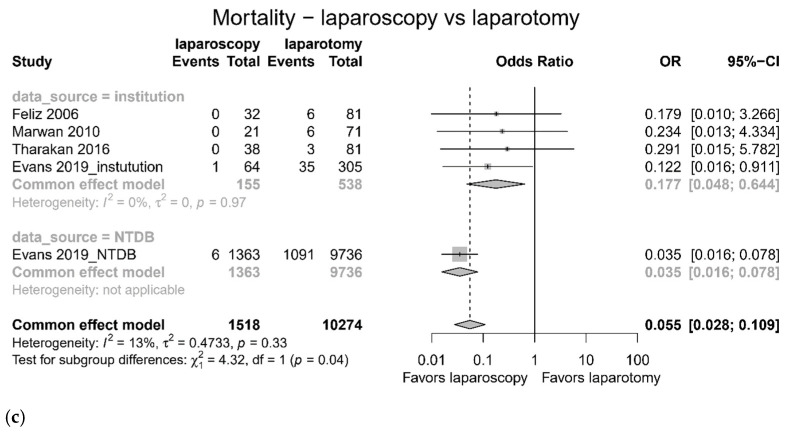
Morbidity and mortality (laparoscopy versus laparotomy). (**a**) Hospital stay, (**b**) complications, and (**c**) mortality.

Meta-analysis showed younger age (SMD −0.47, 95% confidence interval (CI) −0.52 to −0.42, I^2^ = 83%) [19,22,24,25], lower ISS (SMD −0.62, 95% CI −0.67 to −0.57, I^2^ = 97%) [19,21,24,25] (Figure 2), shorter hospital stay (SMD −0.55, 95% CI −0.60 to −0.50, I^2^ = 96%) [19,21,24,25], less complications (OR 0.375, 95% CI 0.309 to 0.455, I^2^ = 67%) [20,22,25], and lower mortality rate (OR 0.055, 95% CI 0.0.28 to 0.109, I^2^ = 13%) [19,21,24,25] in the laparoscopy group compared to the laparotomy group (Figure 3). The pooled operation time did not differ (SMD 0.22, 95% CI −0.23 to 0.27, I^2^ = 93%) [20,22,25] between the laparoscopy and laparotomy groups (Figure 2).

### 3.3. Pooled Incidence of Morbidity and Mortality in Laparoscopy

**Figure 4:**
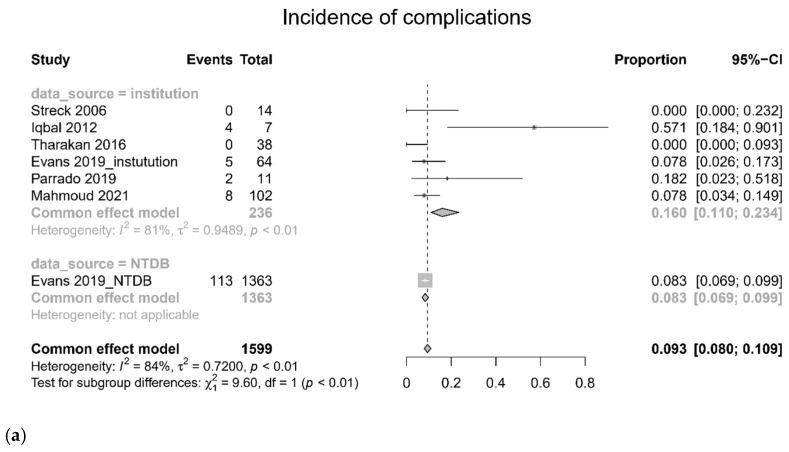

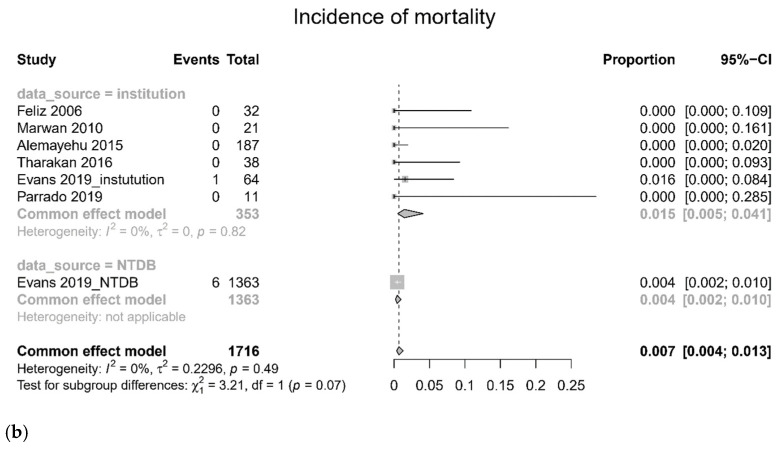
Pooled incidence of morbidity and mortality. (**a**) Complications; (**b**) mortality.

The meta-analysis of the pooled incidence of morbidity and mortality, including non-comparative studies, is summarized in Figure 4. Notably, our meta-analysis showed low complication rate (0.093, 95% CI 0.080 to 0.109, I^2^ = 84%) [20,22,24,25,26,27] and mortality (0.007, 95% CI 0.004 to 0.013, I^2^ = 0%) [19,21,23,24,25,26] in laparoscopy.

### 3.4. Quality of Laparoscopic Procedure

**Figure 5:**
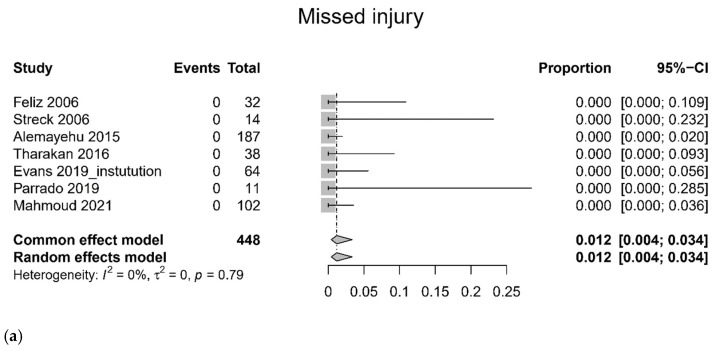

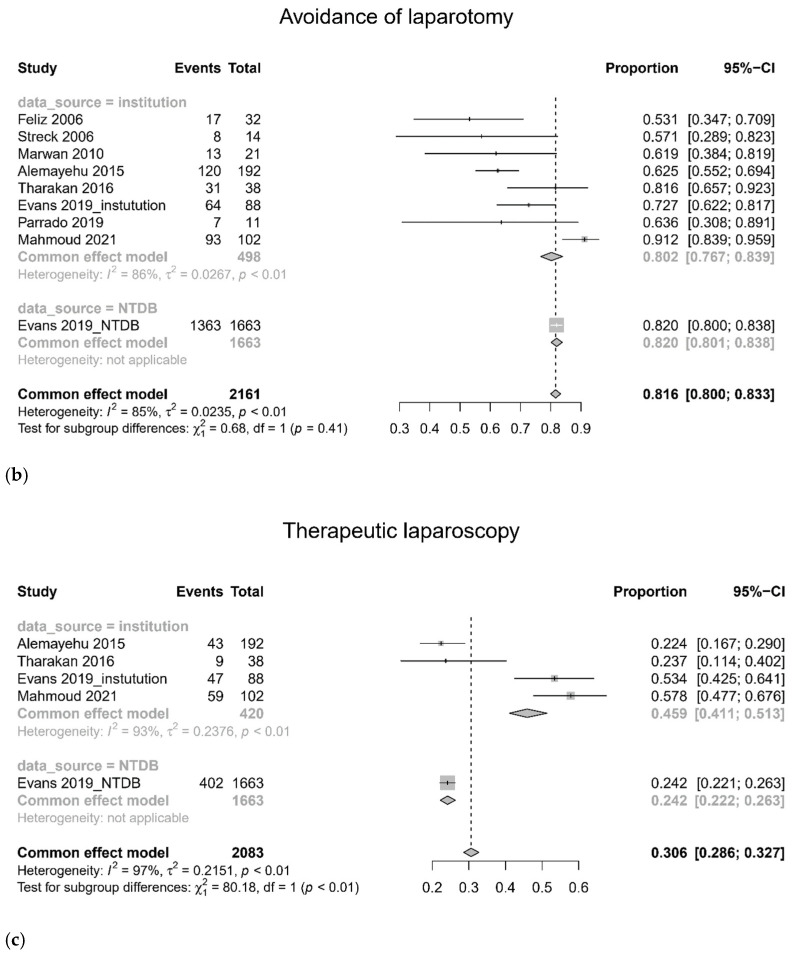
Quality of laparoscopic procedure. (**a**) missed injury, (**b**) avoidance of laparotomy, and (**c**) therapeutic laparoscopy.

There were no missed injuries during the laparoscopic procedure in seven eligible studies [19,20,23,24,25,26,27]. One subset using NTDB [25] did not report any missed injuries. Overall, the majority of patients were able to avoid laparotomy (0.816, 95% CI 0.800 to 0.833, I^2^ = 85%) [19,20,21,23,24,25,26,27], and they underwent a successful laparoscopy. The pooled proportion of therapeutic laparoscopy was 0.306 (95% CI 0.286 to 0.327, I^2^ = 97%) [23,24,25,27] (Figure 5).

### 3.5. Subgroup Analysis and Sensitivity Analysis

We conducted a subgroup analysis according to the data source. In the test for subgroup differences, we found significant statistical differences in terms of hospital stay (laparoscopy vs. laparotomy, *p* < 0.01, Figure 3) [19,21,24,25], mortality (laparoscopy vs. laparotomy, *p* = 0.04, Figure 3) [19,21,24,25], pooled incidence of complications (*p* < 0.01, Figure 4) [20,22,24,25,26,27], and pooled proportion of therapeutic laparoscopy (*p* < 0.01, Figure 5) [23,24,25,27]. In sensitivity analysis after omitting each study, one study that used NTDB data [25] had a significant influence in terms of hospital stay (SMD, omitting NTDB (−0.83, 95% CI −1.02 to −0.64) vs. common effect model (−0.55, −0.60 to −0.50)) and therapeutic laparoscopy (pooled proportion, omitting NTDB (0.46, 95% CI 0.41 to 0.51) vs. common effect model (0.31, 0.29 to 0.33)). The sensitivity analysis, by deleting a study (Iqbal 2012) [22] with pancreatic trauma, showed no significant influence in terms of complications (laparoscopy vs. laparotomy, OR 0.37, 95% CI 0.30 to 0.44, omitting Iqbal 2012 [22]) and incidence of complication (0.083, 95% CI 0.070 to 0.098, omitting Iqbal 2012 [22]). The sensitivity analysis, by deleting a study with liver and spleen injury (Parrado 2019) [26], showed no significant influence in terms of incidence of complication (0.092, 95% CI 0.079 to 0.108, omitting Parrado 2019 [26]), avoidance of laparotomy (0.817, 95% CI 0.800 to 0.833, omitting Parrado 2019 [26]), and incidence of missed injury (0.010, 95% CI 0.003 to 0.029, omitting Parrado 2019 [26]). In terms of other outcomes, there was no significant influence in sensitivity analysis.

### 3.6. Quality Assessment

**Figure 6:**
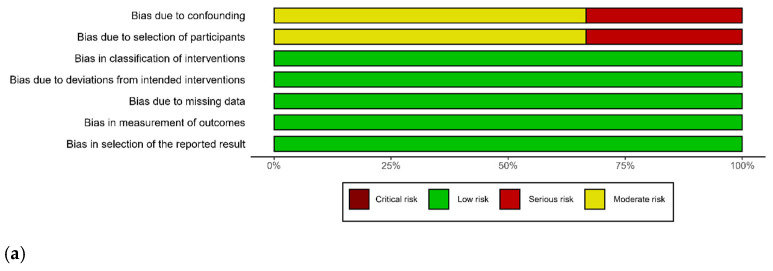

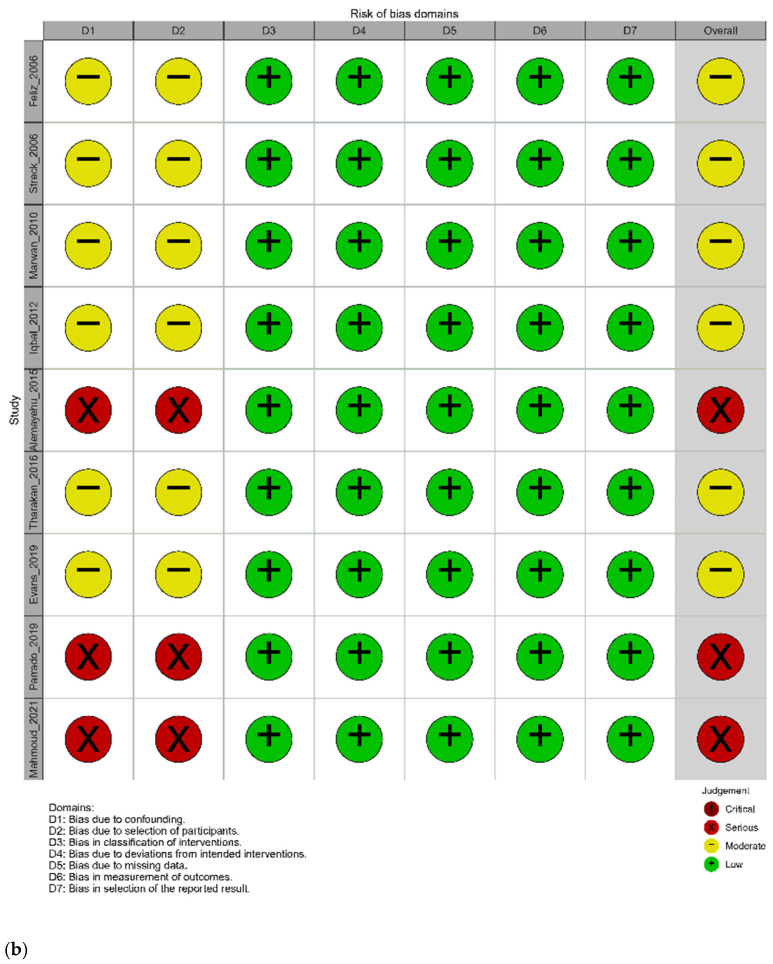
Risk of bias and applicability concerns graph (**a**) and summary (**b**): Review authors’ judgements about each domain presented as percentages across included studies.

All included studies were observational studies. The quality assessment and risk of bias for each eligible study are summarized in Figure 6. Overall, all studies showed moderate-to-serious bias due to the confounding and selection of participants. Injury severity and hemodynamic status were severe in the laparotomy group [19,20,21,22,24,25]. Indeed, non-comparative studies could not measure true effect size compared to laparotomy [23,26,27].

## 4. Discussion

Our analysis demonstrated that laparoscopy in pediatric patients with trauma showed favorable outcomes. The majority of patients could undergo successful laparoscopy without a conversion to open surgery. Indeed, low morbidity and low mortality without missed injuries were observed in the laparoscopy group. To the best of our knowledge, this is the first meta-analysis regarding laparoscopy for pediatric patients with trauma. The evidence regarding pediatric laparoscopy is limited, probably because the incidence of severe trauma is lower in children than in adults [11]. Therefore, our study may provide critical guidance to pediatric laparoscopic procedures. However, a substantial risk of bias was observed, and future prospective studies are warranted.

Currently, the development of laparoscopic skills and equipment enables various surgical procedures equivalent to laparotomy. This development includes high-resolution cameras, useful suturing devices, safe staplers, and excellent energy devices that enable easy and effective resection and anastomosis. In a recent umbrella review of meta-analyses for non-traumatic pediatric patients [31], the authors analyzed 24 meta-analyses regarding different visceral procedures, which included small bowel obstruction repair, anorectal malformation repair, appendectomy, choledochal cyst resection, duodenal obstruction reduction, Kasai portoenterostomy, Ladd’s procedure, pyloromyotomy, and splenectomy. The authors noted that laparoscopy showed a shorter hospital stay, shorter time until full feeding, lower complication rate, lower rate of wound infection, and less intraoperative blood loss [31], thus implying that technical issues are no longer critical. However, in this umbrella review [31], seven meta-analyses showed no advantage of laparoscopy compared with laparotomy regarding duodenal obstruction repair, anorectal malformation repair, appendectomy, fundoplication, Kasai portoenterostomy, and pyloromyotomy. Moreover, laparoscopy showed a longer duration of operation in 16 meta-analyses; [31] hence, the benefits from laparoscopy are still unclear. The operation time did not differ in our meta-analysis between laparoscopy and laparotomy. In particular, in our meta-analysis, we noted that laparoscopy showed no missed injury and high rates in avoiding laparotomy. The most serious concern regarding laparoscopy is the ability of complete exploration of the abdominal cavity and the identification of critical injuries. Moreover, non-therapeutic laparotomy can induce complications, such as postoperative pain, wound infection, incisional hernia, or postoperative small bowel obstruction [2]. Thus, laparoscopy would help surgeons to treat pediatric patients safely and to reduce surgical insults. In our meta-analysis, ISS was lower in the laparoscopy group than in the laparotomy group. This may affect the operation time, hospital stay, complication, and mortality. This selection bias may paradoxically imply that laparoscopy would be more beneficial in selective patients who are stable with mild organ injuries.

In our systematic review, we found several studies using data from NTDB. Among these, only one study [25] comprising 1663 laparoscopies and 9736 laparotomies had large patient numbers and long analysis periods (from 2010 to 2015); thus, we included this study in our meta-analysis. Train et al [30]. reported 160 laparoscopies with 45 open conversions in their study using data from TQIP from 2014 to 2015. Because TQIP utilized the infrastructure of NTDB, it is possible that data from this study may have been duplicated; hence, we excluded it. Swendiman et al. [28]. reported 355 laparoscopies with 66 open conversions (18.6%) and 0.4% mortality in their study with NTDB data from 2010 to 2014. They noted that the use of laparoscopy has increased in pediatric patients with abdominal trauma, typically in patients with mild injuries. They also noted that the increase in utilizing laparoscopy was primarily driven by university hospitals (*p* = 0.026) and level 1 pediatric trauma centers (*p* = 0.043). However, they included only patients younger than 15 years of age. We excluded this study due to duplicability and limited inclusion criteria. Butler et al. [29], in another study using NTDB data, reported 216 laparoscopies and 84 open conversions (38.9%). They found that laparoscopy was associated with shorter hospital stay and a decreased incidence of surgical site infections. However, they included only patients with blunt trauma and excluded severely injured patients, such as patients with hypotension, GCS < 13, ISS > 25, or propensity score <0.05. Indeed, duplicability existed due to the study period (2015 to 2016); therefore, we excluded this study. In excluded studies with NTDB or TQIP data, laparoscopy showed favorable outcomes. Overall, there were a limited number of eligible studies in our review. This may be because severe pediatric trauma is relatively rare compared to adult trauma. Therefore, a study based on nationwide databases with careful design is crucial in order to minimize the bias and to measure the true effect size. In a subgroup analysis of our meta-analysis, we found significant statistical differences in terms of hospital stay and mortality. However, the direction of the effect size was consistent, and laparoscopy showed favorable outcomes in both subgroups. We found lower incidence of therapeutic laparoscopy in the NTDB cohort [25] than the pooled incidence of other studies. However, it was similar to the incidence of therapeutic laparoscopy in two eligible studies [23,24].

Several systematic reviews and meta-analyses regarding adult trauma patients have been reported [1,4,5,6,7]. In our most recent meta-analysis including 19 observational studies regarding laparoscopy for blunt adult trauma [1], laparoscopy showed shortened hospital stay, low morbidity rate, and rare missed injury. In particular, the conversion rate has improved in recent studies, and this may be due to an improvement in laparoscopic skills and devices. In another meta-analysis including 13 prospective and 38 retrospective studies regarding penetrating adult trauma [7], the authors noted 83 missed injuries with 66.7–100% sensitivity and 33–100% specificity. In another recent meta-analysis including 9817 laparoscopies between 1990 and 2016 [6], the authors noted that the incidence of therapeutic laparotomies decreased from 69% to 47.5%, whereas the incidence of therapeutic laparoscopies increased from 7.2% to 22.7%. This may reflect the development of laparoscopic skills and instruments. In adult patients with trauma, laparoscopy showed good outcomes in both blunt and penetrating trauma. However, there have been no previous systematic reviews and meta-analyses regarding pediatric trauma. Thus, as a first systematic review and meta-analysis, our meta-analysis would contribute to a better understanding of laparoscopic surgery and an improvement of surgical outcomes in pediatric patients with trauma.

Our study has several limitations. First, all eligible studies were observational, while no randomized control trials were included. However, this type of study design for pediatric patients would be challenging in clinical practice. The selection bias can arise from different injury severity between laparoscopy and laparotomy. Second, one study that was retrieved from a nationwide registry used heterogenous definitions of interventions, such as open conversion or therapeutic laparoscopy, because this study used the NTDB procedure code to define the intervention while other studies defined it via chart reviews. Thus, we conducted subgroups according to the source of data and conducted sensitivity analysis. Third, one study [22] comprised only pancreatic trauma, which is heterogenous relative to other eligible studies, and this can lead to misreading the results. To overcome this issue, we conducted sensitivity analysis and identified no significant influence. Fourth, we computed pooled incidence by using single descriptive statistics that may induce substantial heterogeneity. To overcome this weakness, we conducted subgroup and sensitivity analyses. Fifth, the analysis of the publication of bias was limited due to the small number of eligible studies, which may induce substantial statistical instability. Finally, we included only articles written in English.

## 5. Conclusions

Laparoscopy for stable pediatric patients showed favorable outcomes in terms of morbidity and mortality. There were no missed injuries, and avoiding laparotomy was possible for the majority of the patients. However, the substantial risk of bias and lack of randomized control trials limits the extrapolation of the results. Nevertheless, laparoscopy appears to be a safe and effective option in selective patients.

## Figures and Tables

**Table 1:** Main characteristics of the eligible studies.

Author	Year	Data Source	Location	Number of Patients	Study Design	Age (Year)	Hemodynamic Status	Indication	Injury Mechanism in Patients with Laparoscopy
Feliz [19]	2006	Single Children’s hospital (2000–2004)	USA	laparoscopy (32), laparotomy (81)	obs, comparative	≤18	Stable	pneumoperitoneum, mesenteric infiltration	Blunt (81%), penetrating (19%)
Streck [20]	2006	Two Children’s hospitals (1998–2003)	USA	laparoscopy (14), laparotomy (36)	obs, comparative	<16	Stable	isolated bowel injury	Blunt (NR), penetrating (NR)
Marwan [21]	2010	Single Children’s hospital (1997–2009)	USA	laparoscopy (21), laparotomy (71)	obs, non-comparative	5 to 15	Stable	pediatric abdominal trauma	Blunt (28.5%), penetrating (71.5%)
Iqbal [22]	2012	Six level-1 PTCs (2000–2010)	USA	laparoscopy (14), laparotomy (7)	obs, comparative	<18	Stable	Grade 3 pancreas injury	NR
Alemayehu [23]	2015	Six PTCs (2000–2010)	USA	laparoscopy (192)	obs, non-comparative	9.6 (4.2)	NR	pediatric abdominal trauma	Blunt (60%), penetrating (40%)
Tharakan [24]	2016	Single level-1 PTC (2000–2012)	USA	laparoscopy (38), laparotomy (81)	obs, comparative	≤18	Stable	pediatric abdominal trauma	Blunt (57.9%), penetrating (42.1%)
Evans [25]	2019	Single level-1 PTC (2005–2017)	USA	laparoscopy (88), laparotomy (305)	obs, comparative	≤18	Stable	pediatric abdominal trauma	Blunt (63%), penetrating (31%)
		NTDB (2010–2015)	USA	laparoscopy (1663), laparotomy (9736)	obs, comparative	≤18	Stable	pediatric abdominal trauma	Blunt (50%), penetrating (44%)
Parrado [26]	2019	Ten level-1 PTCs (2013–2016)	USA	laparoscopy (11)	obs, non-comparative	≤18	Stable	Blunt liver and spleen injury	Blunt (100%)
Mahmoud [27]	2021	Three tertiary pediatric surgery center (2015–2020)	Egypt, Saudi Arabia	laparoscopy (102)	obs, non-comparative	1 to 14	Stable	penetrating pediatric abdominal trauma	Penetrating (100%)

obs, observational; NR, not reported; PTC, pediatric trauma center; NTDB, national trauma database.

## Data Availability

Not applicable.

## References

[1] Ki Y.-J., Jo Y.-G., Park Y.-C., Kang W.-S. (2021). The Efficacy and Safety of Laparoscopy for Blunt Abdominal Trauma: A Systematic Review and Meta-Analysis. J. Clin. Med..

[2] Shamim A.A., Zeineddin S., Zeineddin A., Olufajo O.A., Mathelier G.O., Iii E.E.C., Fullum T., Tran D. (2019). Are we doing too many non-therapeutic laparotomies in trauma? An analysis of the National Trauma Data Bank. Surg. Endosc..

[3] Fredriksson F., Christofferson R.H., Lilja H.E. (2016). Adhesive small bowel obstruction after laparotomy during infancy. Br. J. Surg..

[4] Hajibandeh S., Hajibandeh S., Gumber A.O., Wong C.S. (2016). Laparoscopy versus laparotomy for the management of penetrating abdominal trauma: A systematic review and meta-analysis. Int. J. Surg..

[5] Li Y., Xiang Y., Wu N., Wu L., Yu Z., Zhang M., Wang M., Jiang J., Li Y. (2015). A Comparison of Laparoscopy and Laparotomy for the Management of Abdominal Trauma: A Systematic Review and Meta-analysis. World J. Surg..

[6] Cirocchi R., Birindelli A., Inaba K., Mandrioli M., Piccinini A., Tabola R., Carlini L., Tugnoli G., Di Saverio S. (2018). Laparoscopy for Trauma and the Changes in Its Use from 1990 to 2016: A Current Systematic Review and Meta-Analysis. Surg. Laparosc. Endosc. Percutan. Tech..

[7] O’Malley E., Boyle E., O’Callaghan A., Coffey J.C., Walsh S.R. (2013). Role of Laparoscopy in Penetrating Abdominal Trauma: A Systematic Review. World J. Surg..

[8] Bonjer H.J., Deijen C.L., Abis G.A., Cuesta M.A., Van Der Pas M.H.G.M., de Lange-de Klerk E.S.M., Lacy A.M., Bemelman W.A., Andersson J., Angenete E. (2015). A Randomized Trial of Laparoscopic versus Open Surgery for Rectal Cancer. N. Engl. J. Med..

[9] Nakamura M., Nakashima H. (2013). Laparoscopic distal pancreatectomy and pancreatoduodenectomy: Is it worthwhile? A meta-analysis of laparoscopic pancreatectomy. J. Hepato-Biliary-Pancreat. Sci..

[10] Park Y.K., Yoon H.M., Kim Y.-W., Park J.Y., Ryu K.W., Lee Y.-J., Jeong O., Yoon K.Y., Lee J.H., Lee S.E. (2018). Laparoscopy-Assisted versus Open D2 Distal Gastrectomy for Advanced Gastric Cancer: Results from a Randomized Phase II Multicenter Clinical Trial (COACT 1001). Ann. Surg..

[11] Chang M.C., Chair TQIP Committee (2016). National Trauma Data Bank Report 2016.

[12] Moher D., Liberati A., Tetzlaff J., Altman D.G., the PRISMA Group (2009). Preferred reporting items for systematic reviews and meta-analyses: The PRISMA statement. BMJ.

[13] Wan X., Wang W., Liu J., Tong T. (2014). Estimating the sample mean and standard deviation from the sample size, median, range and/or interquartile range. BMC Med. Res. Methodol..

[14] Sterne J.A.C., Hernán M.A., Reeves B.C., Savović J., Berkman N.D., Viswanathan M., Henry D., Altman D.G., Ansari M.T., Boutron I. (2016). ROBINS-I: A tool for assessing risk of bias in non-randomised studies of interventions. BMJ.

[15] Barendregt J.J., Doi S.A., Lee Y.Y., Norman R.E., Vos T. (2013). Meta-analysis of prevalence. J. Epidemiol. Commun. Health.

[16] Schwarzer G., Chemaitelly H., Abu-Raddad L.J., Rücker G. (2019). Seriously misleading results using inverse of Freeman-Tukey double arcsine transformation in meta-analysis of single proportions. Res. Synth. Methods.

[17] Higgins J.P.T., Thompson S.G., Deeks J.J., Altman D.G. (2003). Measuring inconsistency in meta-analyses. BMJ.

[18] Lau J., Ioannidis J.P.A., Terrin N., Schmid C.H., Olkin I. (2006). The case of the misleading funnel plot. BMJ.

[19] Feliz A., Shultz B., McKenna C., Gaines B.A. (2006). Diagnostic and therapeutic laparoscopy in pediatric abdominal trauma. J. Pediatr. Surg..

[20] Streck C.J., Lobe T.E., Pietsch J.B., Lovvorn H.N. (2006). Laparoscopic repair of traumatic bowel injury in children. J. Pediatr. Surg..

[21] Marwan A., Harmon C.M., Georgeson K.E., Smith G.F., Muensterer O.J. (2010). Use of Laparoscopy in the Management of Pediatric Abdominal Trauma. J. Trauma.

[22] Iqbal C.W., Levy S.M., Tsao K., Petrosyan M., Kane T.D., Pontarelli E.M., Upperman J.S., Malek M., Burns R.C., Hill S. (2012). Laparoscopic Versus Open Distal Pancreatectomy in the Management of Traumatic Pancreatic Disruption. J. Laparoendosc. Adv. Surg. Tech..

[23] Alemayehu H., Clifton M., Santore M., Diesen D., Kane T., Petrosyan M., Franklin A., Lal D., Ponsky T., Nalugo M. (2015). Minimally Invasive Surgery for Pediatric Trauma—A Multicenter Review. J. Laparoendosc. Adv. Surg. Tech..

[24] Kim A.G., Collins J.L., Nance M.L., Blinman T.A., Tharakan S.J. (2016). Laparoscopy in Pediatric Abdominal Trauma: A 13-Year Experience. Eur. J. Pediatr. Surg..

[25] Evans P.T., Phelps H.M., Zhao S., Van Arendonk K.J., Greeno A.L., Collins K.F., Lovvorn H.N. (2020). Therapeutic laparoscopy for pediatric abdominal trauma. J. Pediatr. Surg..

[26] Parrado R., Notrica D.M., Garcia N.M., Alder A.C., Eubanks I.J., Maxson R.T., Letton R.W., Ponsky T.A., Peter S.D.S., Leys C. (2019). Use of Laparoscopy in Pediatric Blunt and Spleen Injury: An Unexpectedly Common Procedure after Cessation of Bleeding. J. Laparoendosc. Adv. Surg. Tech..

[27] Mahmoud M.A., Daboos M.A., Bayoumi A.S.S., Helal A.A., Almaawi A., Hassab M.H., Aldaraan K.Z. (2021). Role of Minimally Invasive Surgery in Management of Penetrating Abdominal Trauma in Children. Eur. J. Pediatr. Surg..

[28] Swendiman R.A., Goldshore M.A., Blinman T.A., Nance M.L. (2019). Laparoscopic Management of Pediatric Abdominal Trauma: A National Trauma Data Bank Experience. J. Laparoendosc. Adv. Surg. Tech..

[29] Butler E.K., Mills B.M., Arbabi S., Groner J.I., Vavilala M.S., Rivara F.P. (2020). Laparoscopy Compared with Laparotomy for the Management of Pediatric Blunt Abdominal Trauma. J. Surg. Res..

[30] Train A.T., Naseem H.-U., Chen Z., Wilding G.E., Bass K.D., Noyes K., Train W.W., Rothstein D.H. (2019). Predictors and Outcomes of Laparoscopy in Pediatric Trauma Patients: A Retrospective Cohort Study. J. Laparoendosc. Adv. Surg. Tech..

[31] Kuebler J.F., Dingemann J., Ure B.M., Schukfeh N. (2020). Thirty Years of Minimally Invasive Surgery in Children: Analysis of Meta-Analyses. Eur. J. Pediatr. Surg..

